# Rules of Business

**Published:** 1884-03

**Authors:** 


### SELECTIONS.

### Rules of Business.

My mother was an extensive milliner, and became quite popular. One day, as I was approaching manhood, she advised me in substance as follows:

My son, it is for you to say whether, as a business man, you will be a success or a failure. The majority of men in all businesses are failures. But I think if you will carry out the following rules you may be successful:

1st. Be thoroughly qualified for your business. For success do not trust chance, outward show, or assumption. Merit alone will give permanent prosperity.

2d. Go to the people, and not expect the people to come to you. An out of the way place of business is dear at any price. If you are well qualified for what you do, it will pay you to have a good location though you have to pay well for it. But even there you cannot afford to fold your hands and expect the people to find you out. By all legitimate means let them see who you are and what you can do. The charlatan advertises and shows himself a charlatan; and there are ways for a business man to advertise and show himself to be a business man. As a professional man avoid advertising as you would avoid quackery—that is, advertising in the ordinary sense of that term. Yet, advertise. Make it an essential part of your business. There are ways of advertising and yet not advertising; being prominently before the community, and yet give no offence, but attract by what you say. You may do great good by imparting solid truths and timely suggestions, and showing an interest in their welfare from your professional standpoint. If this is done with great caution, modesty, and good judgment, you will obtain their hearty thanks and all the support you deserve.

3d. Make your place of business attractive. Not gaudy, or even showy, but yet rich and homelike—well-appointed, and yet putting your customers or patients at their ease by its inviting aspect. Do not say you cannot afford it. You cannot, and should not if you could, unless your qualifications, culture, and dignity correspond; but if you really belong there, and will take the proper course to maintain yourself in it, Mr. Public Opinion will pay the bills and leave you a good profit.

4th. Receive all with gentlemanly courtesy and frankness. Avoid the two extremes of great familiarity and unapproachable reserve. Let your whole demeanor show that you are the master of your situation, and that at the same time you are eminently a fellow with those you serve—sympathizing with their troubles, interested in their wants, and determined to please them in your services. Study the difference between pity and sympathy, pedantry and intelligence, foolishness and affability, austerity and dignity.

5th. Maintain your moral integrity. Be conservative without yielding principle; seek to please without compromising the right; win by your graces without losing your self-respect, and be in the community a positive power in every good cause.

6th. Work for the future. Slighted work, erronious advice, and yielding to popular prejudices may give good financial showing for the day, but their effect upon the future will be damaging indeed. Better be reckoned a slow workman than a poor one; better maintain an unpopular course than a wrong one; better be a little ahead of the masses and receive their jeers than to stultify conscience and receive applause. By and by they will award you the greater honors.

7th. Remember you will never be too old to study, and to improve in all the elements of wisdom and skill. The world is moving forward, and if you would keep up with it, you must be able to do to-worrow what you can not do to-day; and know more both concerning your specific calling and of the world in general.—*Items of Interest.*

---

During the performance of a thrilling play in Norwalk, Conn., recently, a well-known physician of the town, who seldom entered a theatre, was among the audience. When the part came where the heroine swallowed the poison, is dying, and her lover and friends are wringing their hands and crying, helplessly, "What can be done?" the doctor was seen to be laboring under considerable excitement. At last the terrible scene was too much for him, and, forgetting where he was, he jumped to his feet and shouted: "Give her coffee, you fools; give her coffee!" Then a friendly hand pulled him back into his seat, and the surprised actors went on with the play.

---

A Dental Institute is to be added to the Berlin University, and plans for its organization have been submitted to the medical faculty. The consideration in which American dentists who have established themselves in European cities are held, and the prices which they are able to charge, furnish sufficient proof that such an institution is needed in other countries besides Germany.

